# Assessment of the In Vitro Cytotoxic Profile of Two Broad-Spectrum Antibiotics—Tetracycline and Ampicillin—On Pharyngeal Carcinoma Cells

**DOI:** 10.3390/medicina58091289

**Published:** 2022-09-16

**Authors:** Daniel Florin Pancu, Robert Cosmin Racea, Ioana Macasoi, Cristian Andrei Sarau, Iulia Pinzaru, Marioara Poenaru, Laura-Cristina Rusu, Cristina Adriana Dehelean, Stefania Dinu

**Affiliations:** 1 Faculty of Medicine, “Victor Babes” University of Medicine and Pharmacy Timisoara, Eftimie Murgu Square No. 2, 300041 Timisoara, Romania; 2 Faculty of Dental Medicine, “Victor Babes” University of Medicine and Pharmacy, 9 Revolutiei 1989 Ave., 300070 Timisoara, Romania; 3 Multidisciplinary Center for Research, Evaluation, Diagnosis and Therapies in Oral Medicine, “Victor Babes” University of Medicine and Pharmacy, Eftimie Murgu Square, No. 2, 300041 Timisoara, Romania; 4 Faculty of Pharmacy, “Victor Babes” University of Medicine and Pharmacy Timisoara, Eftimie Murgu Square No. 2, 300041 Timisoara, Romania; 5 Research Center for Pharmaco-Toxicological Evaluations, Faculty of Pharmacy, “Victor Babes” University of Medicine and Pharmacy Timisoara, Eftimie Murgu Square No. 2, 300041 Timisoara, Romania; 6 Department of Oral Pathology, Multidisciplinary Center for Research, Evaluation, Diagnosis and Therapies in Oral Medicine, “Victor Babes” University of Medicine and Pharmacy Timisoara, 2 Eftimie Murgu Sq., 300041 Timisoara, Romania; 7 Pediatric Dentistry Research Center, Faculty of Dental Medicine, “Victor Babes” University of Medicine and Pharmacy Timisoara, No. 9, Revolutiei Bv., 300041 Timisoara, Romania

**Keywords:** tetracycline, ampicillin, pharyngeal cells, rhodamine-phalloidin staining, DAPI staining

## Abstract

*Background and Objectives:* In spite of the fact that antibiotics are considered to be the cornerstone of modern medicine, their use in the treatment of cancer remains controversial. In the present study, the main objective was to examine the effects of two antibiotics—tetracycline and ampicillin—on the viability, morphology, migration, and organization and structure of the nuclei and the actin fiber network of pharyngeal carcinoma cells—Detroit-562. *Materials and Methods:* In order to determine the viability of the cells, the 3-(4,5-dimethylthiazol-2-yl)-2,5-diphenyltetrazolium bromide (MTT) method was applied after the cells were stimulated with five concentrations of tetracycline and ampicillin (10, 25, 50, 75, and 100 μM) for 72 h. A scratch assay was used to assess the migration ability of the cells. For the visualization of the nuclei and actin fibers, 4,6-diamidino-2-phenylindole (Dapi) and Rhodamine-Phalloidin were used. *Results:* There are different effects of tetracycline and ampicillin. Thus, tetracycline: (i) exhibited a concentration-dependent cytotoxic effect, decreasing cell viability to approximately 46%; (ii) inhibits cellular migration up to 16% compared to 60% for control cells; and (iii) induces changes in cell morphology as well as apoptotic changes in the nucleus and F-actin fibers. In contrast, in the case of ampicillin, an increase in viability up to 113% was observed at 10 μM, while a decrease in viability up to approximately 94% was observed at the highest concentration tested (100 μM). *Conclusions:* The results indicated a different effect regarding the impact on pharyngeal carcinoma cells. Thus, tetracycline has a concentration-dependent cytotoxic effect, while in the case of ampicillin a slight stimulation of cell viability was observed.

## 1. Introduction

In terms of incidence, head and neck cancer ranks sixth, and almost 50% of cases are oral cancers [1]. While significant progress has been made in the management and treatment of these cancers, the 5-year survival rate for oral cancer still falls below 50% in most countries. In light of the heterogeneity of the cancers of the head and neck, treating these conditions can be challenging [2]. Many factors contribute to the development of oral cancer, including smoking, exposure to UV radiation, chronic inflammation, and certain bacterial or viral infections [3]. Treatment options for oropharyngeal cancers include chemotherapy, radiotherapy, and surgery. An immunotherapy approach to treating these cancers is the most recent treatment option [4].

An important problem associated with antitumor therapy is the occurrence of toxic reactions both locally and systemically. In some cases, chemotherapy and radiation may lead to the need for antibiotic therapy. The majority of antibiotics used in this case are broad-spectrum antibiotics, which increase the risk of altering the physiological microbiome [5]. In recent years, research has been focused on the role of microbiota in pathogenesis and treatment response in a wide variety of diseases, including cancer [6,7]. Thus, a recent estimate showed that approximately 20% of cancers globally are closely related to the microbiota [8]. It has been documented in the literature that intestinal microbiota plays a significant role in gastric cancer development [9]. In contrast, the relationship between oral dysbiosis and oral cancer is not fully understood [10]. It has been proposed in the literature that microbial influence on the cancer process may be mediated through different mechanisms of action, of which the most recognized mechanism is the induction of chronic inflammation [11,12]. As well as this mechanism of action, bacteria may also influence the process of cell proliferation and inhibit cell apoptosis, thus contributing to the development of several types of cancer [13,14]. In terms of throat cancer, such as pharyngeal cancer, there is little evidence regarding the role of microbiota. According to Wang et al., a substantial difference was found between the microbiota of patients with throat cancer and that of healthy patients, emphasizing the important role played by the microbiota in this type of cancer [15]. Furthermore, Gong and colleagues identified similar differences between the microbiota of throat cancer patients and those of healthy individuals [16].

Antibiotics are considered to be the cornerstones of modern medicine, but currently, they are associated with problems related to the resistance of bacteria and, consequently, their inefficiency [17]. Furthermore, antimicrobial resistance can also be associated with increased virulence and transmission, playing a crucial role in the global spread of resistant bacteria [18]. A critical role in maintaining the state of health at the tissue level is played by the homeostasis between the human microbiome and the host. In this way, the virulence of the bacteria can disrupt the homeostasis of the organism and may result in the development of oral tumors [19]. Additionally, recent studies have shown that some antibiotics can be useful in the treatment of cancer by: (i) promoting apoptosis in cells; (ii) inhibiting proliferation of cell lines; and (iii) preventing metastasis [20]. Consequently, antibiotics are currently considered a viable therapeutic option for cancer patients. In contrast, certain antibiotics may disrupt the microbiome, affecting beneficial bacteria such as *Lactobacillus* and *Bifidobacterium* [21]. In light of the fact that the microbiome plays a critical role in the treatment of cancer, these antibiotics may reduce the immune capacity of the body, causing inflammation and reducing the effectiveness of the treatment [22].

Tetracyclines were discovered in the 1930s and were introduced into therapeutic use in 1948. It includes substances that have a broad spectrum of activity, and their antimicrobial activity arises as a result of their ability to inhibit the synthesis of bacteria’s proteins [23]. Tetracycline has a bacteriostatic action, entering the bacterial cell both by passive diffusion and active transport. An important mechanism underlying antibacterial resistance is the efflux system, which also plays a role in virulence. The relationship between resistance and virulence mechanisms, however, has not been extensively studied [24]. Thus, in a study carried out in the case of infants in the first year of life, it was highlighted the fact that 12% of them had strains of *E. coli* resistant to tetracycline, although the children were not exposed to this antibiotic. In the case of resistant *E. coli* strains, genes encoding efflux pumps were identified, carrying virulence genes for *P. fimbriae* and aerobactin. Thus, even in the absence of antibiotic exposure, a large percentage of strains express resistance mechanisms as well as different virulence genes [25]. *Salmonella* genomic island 1 is another example of an efflux pump-mediated resistance mechanism, which contains between 6 and 9 virulence determinants as well as a multidrug resistance region [26]. During the 1980s, the hypothesis of the use of tetracyclines in anti-tumor therapy was promoted on the basis that mitochondrial ribosomes have an evolutionary relationship with bacterial ribosomes [27]. There are several common mechanisms related to tetracyclines that affect tumors, including the inhibition of mitochondrial protein synthesis, inhibition of matrix metalloproteinases, inhibition of nuclear factor kappa (NF-kB) signaling, as well as reduced activity of transcription factors such as Twist I and II, SNAI I and II, STAT3, etc. Tetracyclines also inhibit the formation of new blood vessels [28].

Amplification of the spectrum of activity of penicillins was accomplished by the introduction of ampicillin into medical practice in order to overcome the problem of *S. aureus* resistance to penicillins. Another advantage of ampicillin is its resistance to gastric juice, which makes it suitable for oral administration [29]. As part of its antibacterial action mechanism, ampicillin binds to specific receptors (membrane-bound penicillin-binding proteins) and affects the vital functions of bacteria, such as the formation of morphogenetic processes and alterations of the peptidoglycan structure of the cell wall [30]. A variety of mechanisms are involved in the acquisition of resistance to β-lactams, implicitly to ampicillin, including mutations in penicillin-binding proteins, the production of β-lactamases, and changes in permeability [31]. A relevant example of a virulent strain resistant to ampicillin is the epidemic-virulent clonal complex of *Enterococcus faecium* 17, associated with most hospital outbreaks and clinical infections. In addition to resistance to ampicillin, this complex is characterized by the presence of an island of presumed pathogenicity [32]. There is considerable controversy surrounding the use of ampicillin in cancer patients. Despite the fact that ampicillin can be used prophylactically in oncological patients, it is unknown precisely what effect it may have on the development and proliferation of tumor cells [33]. Hence, there is conflicting evidence regarding the relationship between ampicillin and tumorigenesis. On the one hand, some studies provide evidence that ampicillin has antitumor properties [34]. However, there is evidence in the literature that ampicillin has a pro-tumor effect, leading to the stimulation of tumor cell proliferation, increasing tumor size [35].

Taking into account the hypothesis that broad-spectrum antibiotics have a detrimental effect on the human microbiota, and the pharyngeal carcinoma can be affected by such changes, the main objective of this study was to assess the effects of ampicillin and tetracycline on Detroit-562 pharyngeal carcinoma cells. It was assessed whether the treatment had an effect on viability, morphology, cell migration, and the structure of the nucleus and actin fibers.

## 2. Materials and Methods

### 2.1. Reagents

Two antibiotics were the subjects of the present study, namely Tetracycline (87128-25G), which was purchased from Sigma Aldrich, Merck KgaA (Darmstadt, Germany) and Ampicillin (P60-083910), which was purchased from PAN Biotech (Aidenbach, Germany).

For in vitro studies, the following reagents were used: trypsin-EDTA solution, dimethyl sulfoxide (DMSO), fetal bovine serum (FBS), penicillin/streptomycin, and Cell Growth Determination kit, 3-(4,5-dimethylthiazol-2-yl)-2,5-diphenyltetrazolium bromide MTT-based, obtained from Sigma Aldrich, Merck KgaA (Darmstadt, Germany). All reagents were of analytical grade of purity and for cell culture use.

### 2.2. Cell Culture

In vitro experiments were performed on Detroit-562—pharyngeal carcinoma cells obtained from the American Type Culture Collection (ATCC, code number CCL-138^TM^, LGC StandardsGmbH, Wesel, Germany). The cells were cultured in Eagle’s Minimum Essential Medium (EMEM—ATCC^®^ 30-2003^TM^), supplemented with 10% Fetal Bovine Serum and 1% antibiotic mixture (100 U/mL penicillin/100 g/mL streptomycin). The cells were maintained under standard temperature (37 °C) and 5% CO_2_.

Specifically, Detroit-562 is an adherent cell line isolated from the pharynx of a patient with pharyngeal cancer that has been used extensively for tumor research [36]. To prepare the cells to test the cell viability, several steps were taken, namely: (i) the cells were cultured in T75 cell culture flask, and after reaching a confluence of approximately 70–80%, the cells were washed with phosphate saline buffer (PBS); (ii) PBS was removed from the plate and a volume of 3 mL trypsin-EDTA solution was added, and the cells were incubated for 3 min at 37 °C; (iii) after this time interval, the percentage of cell detachment from the plate is checked, and then, the cell suspension was transferred into a 15-mL conical tube; (iv) the cell suspension was centrifuged at 2500 rpm for 5 min; (v) after centrifugation, the supernatant is removed, and the cell sediment is resuspended in 3–4 mL of fresh medium; and (vi) finally, the cells are counted and cultivated in 96-well plates.

### 2.3. Cellular Viability Assessment

For viability assessment, cells were cultured in 96-well plates with 1 × 10^4^ cells per well. Five concentrations of tetracycline and ampicillin (10, 25, 50, 75, and 100 μM) were added to the cells after they had reached a suitable confluence of 90% for a period of 72 h. In order to determine viability, the culture medium was replaced with a fresh one, in a volume of 100 μL per well, before the viability determination. The MTT solution reagent was added to each well in a volume of 10 μL, and the plates were incubated at 37 °C for 3 h. A solubilization solution of 100 μL per well was then added and the plates were stored at room temperature for 30 min. Using the Cytation 5 (BioTek Instruments Inc., Winooski, VT, USA), absorbents were measured at two wavelengths of 570 nM and 630 nM. The results obtained were expressed as a percentage (viable cells%).

### 2.4. Cellular Morphology

For a more detailed understanding of the effect of the two antibiotics on pharyngeal carcinoma cells, the morphology of the cells was examined after 72 h. Therefore, following these intervals, the morphology of the cells was evaluated microscopically by photographing them under bright field illumination. The images were captured using an Olympus IX73 inverted microscope provided with DP74 camera photo and analyzed with CellSens V1.15 software (Olympus, Tokyo, Japan)

### 2.5. Wound Healing Assay

To analyze the impact of the two antibiotics on pharyngeal carcinoma cells’ migration capacity, a scratch assay was performed. Cells were cultured in Corning plates of 24 wells at a density of 1 × 10^5^ cells/well. The AutoScratch^TM^ Wound Making Tool provided by BioTek^®^ Instruments Inc., Winooski, VT, USA was used to make an automatic scratch after a suitable confluence was achieved. Afterward, the cells were treated for 24 h with 10, 50, and 100 μM concentrations of tetracycline and ampicillin. As a part of the analysis and interpretation of the results, Cytation 1 was used to take photos at the start (0 h) and end of the experiment (24 h), followed by analysis using Gen5 TM Microplate Data Collection and Analysis Software version 3.10 (BioTek^®^ Instruments Inc., Winooski, VT, USA). Calculating the effect of these two antibiotics on cell migration and expressing the results as percentages were accomplished by applying the calculation method previously described in the literature [37].

$$Scratch\;Closure\;After\;24\;h=\frac{Scratch\;Surface\;(0\;h)-Scratch\;Surface\;(24\;h)}{Scratch\;Surface\;(0\;h)}$$

### 2.6. Fluorescence Immunocytochemistry

The cells were cultured in 12-well plates at a density of 1 × 10^5^ cells per well in order to determine the impact on actin fibers and the nucleus. Following the cell confluence of approximately 90%, the cells were stimulated with three concentrations (10, 50, and 100 μM) of ampicillin and tetracycline for a period of 72 h. Once the cells had been washed with ice-cold PBS, they were fixed with 4% paraformaldehyde for 30 min at 4 °C. After fixation, the cells were permeabilized with 2% Triton X-100/1× PBS, and then blocked with 30% FCS/0.01% Triton X-100. Incubation of the cells was performed for 20 min at room temperature, with Rhodamine Phalloidin (00027) from Biotium (Hayward, CA, USA) protecting them from light. Finally, 4,6-diamidino-2-phenylindole (DAPI) was added for 15 min in order to visualize the nuclei.

### 2.7. Statistical Analysis

All data are expressed as means ± standard deviation (SD), the differences being compared by applying the one-way ANOVA analysis followed by Dunnett’s multiple comparisons post-test. The used software was GraphPad Prism version 9.0.0 for Windows (GraphPad Soft-ware, San Diego, CA, USA, www.graphpad.com). The statistically significant differences between data were labeled with * (* *p* < 0.1; ** *p* < 0.01; *** *p* < 0.001; **** *p* < 0.0001).

## 3. Results

### 3.1. Cellular Viability Assessment

The MTT method was used to assess the viability of pharyngeal carcinoma cells after 72 h during which five concentrations of tetracycline and ampicillin (10, 25, 50, 75, and 100 μM) were applied.

Based on the results, tetracycline and ampicillin affect cell viability in different ways. Accordingly, tetracycline causes a decrease in cell viability directly proportional to its concentration. The viability of the cells decreased to approximately 71% at a concentration of 10 μM, while at a concentration of 100 μM the viability declined to approximately 46%.

**Figure 1:**
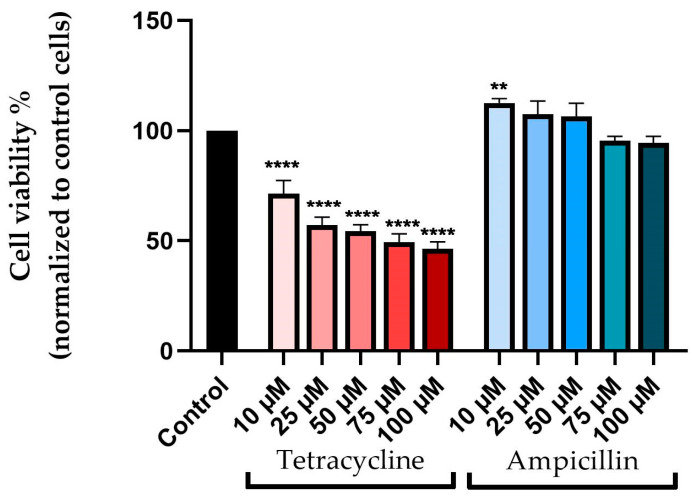
In vitro evaluation of the viability of pharyngeal carcinoma cells—Detroit-562—after stimulation with tetracycline and ampicillin (10, 25, 50, 75, and 100 uM) for 72 h following treatment. The results are expressed as viability percentages (%) normalized to control cells (unstimulated) and expressed as mean values ± SD of three independent experiments performed in triplicate. The statistical difference between the control group and the tetracycline and ampicillin-treated group was achieved by applying one-way ANOVA analysis, followed by Dunnett’s multiple post-test comparisons (** *p* < 0.01; **** *p* < 0.0001).

Ampicillin had a different effect on cell viability than tetracycline when it came to its effect on cell viability. Thus, each of the three lowest concentrations (10, 25, and 50 μM) showed higher cell viability than the unstimulated control cells, with values of 112%, 107%, and 106%, respectively. The viability of cells suffered a slight, but not significant decrease at concentrations of 75 and 100 μM, registering approximately 95% at 75 μM and 94% at 100 μM, respectively (Figure 1).

### 3.2. Cellular Morphology

To gain a deeper understanding of the effects of the two antibiotics on pharyngeal carcinoma cells, the morphology of the cells was analyzed.

**Figure 2:**
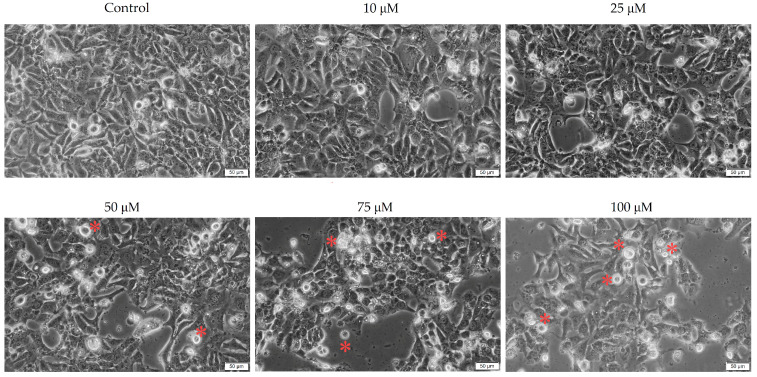
In vitro morphological changes of Detroit-562 cells after 72 h of stimulation with tetracycline (10, 25, 50, 75, and 100 μM). Asterisks indicate morphological changes. The pictures were taken at a magnification of 20×, and the scale bar indicates 50 μm.

Tetracycline causes morphological changes and a decrease in cellular confluence in a concentration-dependent manner. Morphological changes were observed at all concentrations studied, including (i) cell rounding, (ii) cell detachment from plaques, and (iii) confluence and cell number decreases. The effects of the 100 μM concentration on cell morphology were the most evident (Figure 2).

**Figure 3:**
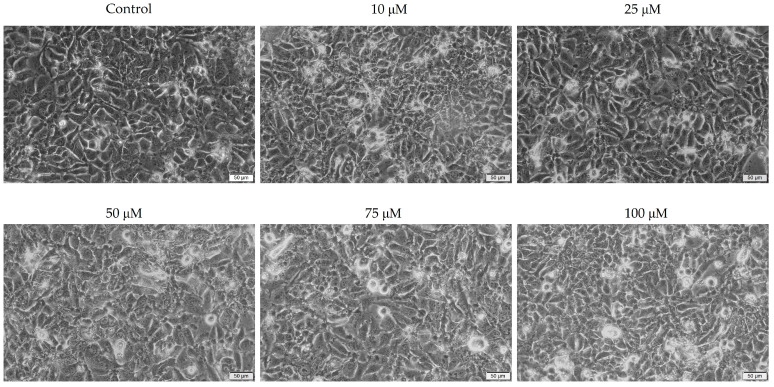
In vitro morphological changes of Detroit-562 cells after 72 h of stimulation with ampicillin (10, 25, 50, 75, and 100 μM). The pictures were taken at a magnification of 20×, and the scale bar indicates 50 μm.

Conversely, in the case of ampicillin, there was no significant morphological damage compared to the control cells. No noticeable decrease in the number and confluence of cells was observed in this case, both of which remained relatively constant at all concentrations evaluated. These results are consistent with those obtained previously in the assessment of cell viability (Figure 3).

### 3.3. Wound Healing Assay

In view of the fact that cell migration is a characteristic of tumor cells, the present study evaluated the impact of tetracycline and ampicillin tested in three concentrations (10, 50, and 100 μM) on cell migration.

**Figure 4:**
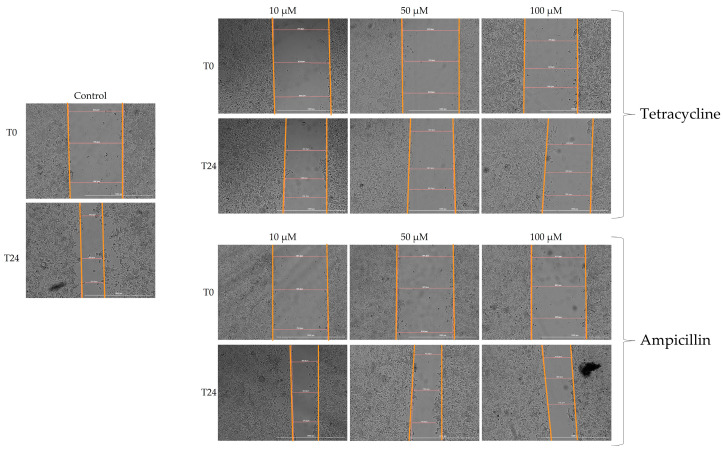
In vitro scratch wound healing image of the Detroit-562. The images were taken at the beginning of the experiment (T0) and after 24 h of stimulation (T24). The pictures were taken at a magnification 10×, and the scale bar indicates 1000 µm.

**Figure 5:**
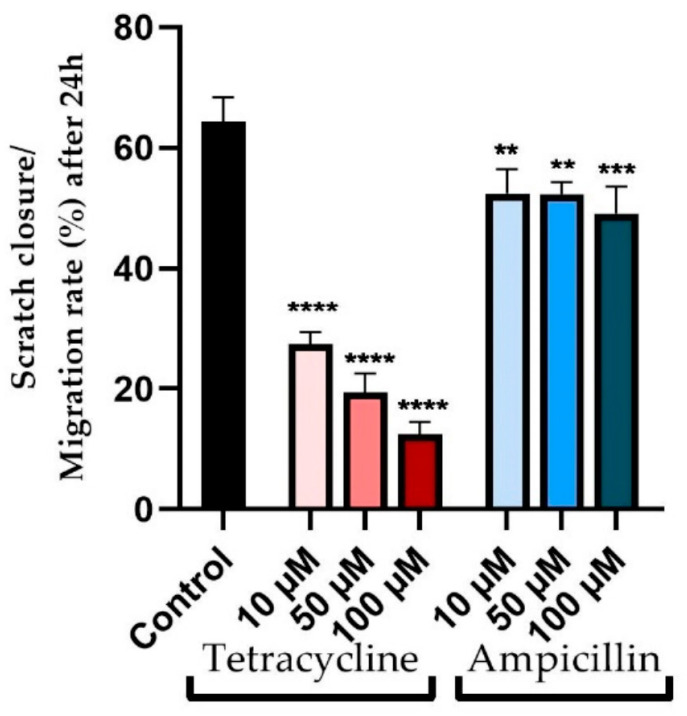
Quantitative evaluation of cell migration expressed as a percentage compared to control (non-stimulated) cells. The results are expressed as mean values ± SD of three independent experiments performed in triplicate. The statistical differences between control and cells stimulated with tetracycline and ampicillin were determined by applying the one-way ANOVA analysis followed by the Dunnett’s multiple comparisons post-test (** *p* < 0.01; *** *p* < 0.001 and **** *p* < 0.0001).

The effects of tetracycline were evident in the inhibition of cell migration as well as morphological changes (Figure 4). There was a correlation between the tested concentration and the decrease in cell migration capacity. As a result, at a concentration of 10 μM, the closure rate decreased to approximately 27%, compared to approximately 64% for control cells. In the case of concentrations of 50 and 100 μM, greater decreases in cell migration were observed, with a reduction in approach rates of 19% and 12%, respectively (Figure 5).

The effect of ampicillin on cell migration, however, was not as severe as that of tetracycline, and also the morphology of the cells was not significantly affected (Figure 4). At concentrations of 10 and 50 μM, similar approach rates of approximately 52% were observed. Slight inhibition of cell migration was observed at the highest tested concentration of 100 μM, with an approach rate of approximately 49% (Figure 5).

### 3.4. Fluorescence Immunocytochemistry

As a more comprehensive picture of the mode of action of the two antibiotics at the cellular level, fluorescence immunocytochemistry was used to observe changes at the level of actin fibers and the nucleus following stimulation with tetracycline and ampicillin in three concentrations—10, 50, and 100 μM for 72 h.

**Figure 6:**
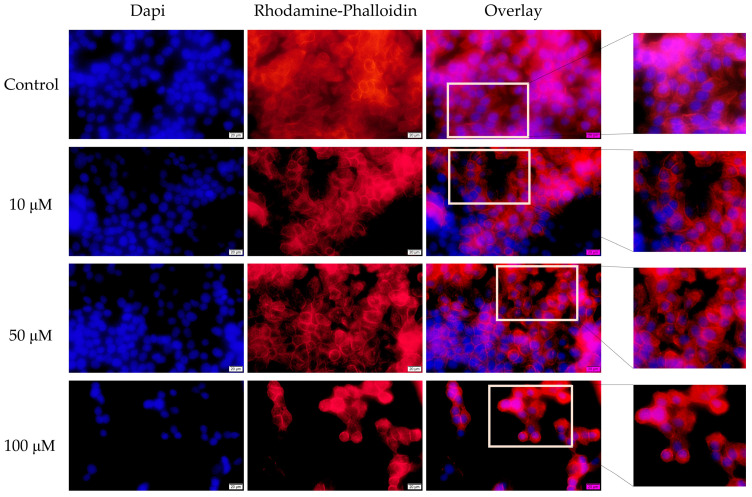
The impact of tetracycline (10, 50, and 100 μM) after 72 h at the level of: nuclei—DAPI staining (blue) and F-actin fibers—Rhodamine Phalloidin (red). The pictures were taken using a 40× objective at a scale bar of 10 µm.

Actin fibers were visualized using Rhodamine-Phalloidin staining. A change in the distribution of actin fibers was observed in the cells treated with tetracycline compared with control cells. Throughout the cells, actin fibers were highly concentrated at the edges, indicating a condensed state. While unstimulated cells had uniform distributions of actin fibers throughout the entire cell. Regarding the effect on the nuclei, tetracycline caused a strong condensation of chromatin, the appearance of apoptotic bodies, and a decrease in the number of nuclei. Concentrations of 100 μM resulted in the most significant changes (Figure 6).

**Figure 7:**
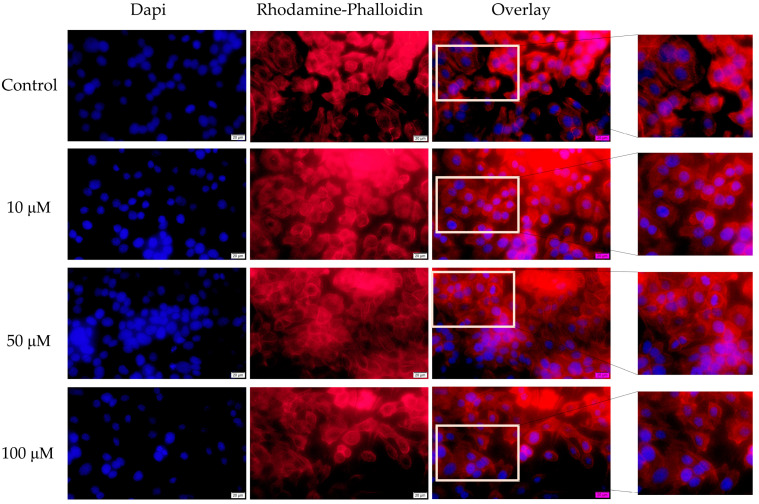
The impact of ampicillin (10, 50, and 100 μM) after 72 h at the level of: nuclei—DAPI staining (blue) and F-actin fibers—Rhodamine Phalloidin (red). The pictures were taken using a 40× objective at a scale bar of 10 µm.

Contrary to tetracycline, the changes to actin fibers attributable to ampicillin were not as significant as those attributable to tetracycline. In the results, actin fibers were found to have a uniform distribution similar to that observed in control, unstimulated cells. At the same time, only a slight condensation of chromatin was observed in the nuclei after the treatment at 100 μM (Figure 7).

## 4. Discussion

Today, pharyngeal cancer is a major problem faced by mankind, largely because of alcohol consumption and smoking [38]. There has been considerable interest in examining the causal relationship between the composition of the microbiota and the development of cancer. As a result, it has been demonstrated that different changes in microbiota are associated with the development of various types of cancer [15]. There has been evidence that colonization with salivary bacteria such as *Porphyromonas gingivalis* and *Fusobacterium nucleatum* can lead to the development of oral cancer [39,40]. There is a lack of knowledge regarding how the microbiome contributes to tumor development. Several studies have confirmed the involvement of different microbes in promoting carcinogenesis by producing carcinogenic substances and immunosuppressive substances [41,42,43]. According to Frank and colleagues, the administration of antibiotics prevented or delayed the induction of the murine aryl hydrocarbon receptor (Ahr) pathway, suggesting a possible association between dysbiosis and head and neck cancers [44]. It is well known that AhR activation plays a critical role in both autoimmune diseases as well as various types of cancer, including head and neck cancer, where it promotes the migration and proliferation of tumor cells [45,46,47]. Moreover, studies conducted on mice demonstrated a link between the administration of antibiotics active against Gram-positive bacteria and the effectiveness of antitumor therapy [48,49].

Radiation therapy and chemotherapy are the most common therapeutic strategies in the treatment of pharyngeal cancer. There is, however, a risk of toxic reactions associated with conventional therapy, which may require the use of broad-spectrum antibiotics [5]. There is controversy surrounding the use of antibiotics in the treatment of cancer patients. Aside from altering the microbiota, antibiotics also reduce immunity and promote inflammation, which contributes to tumor development and decrease treatment effectiveness [50]. Several antibiotics with antitumor activity have also been discussed in the literature, which are known to have a strong anti-proliferative effect on tumor cells [51]. In light of these premises, the current study investigated the effects of two antibiotics, namely tetracycline and ampicillin, on pharyngeal carcinoma cells. Therefore, the viability and morphology of the cells, as well as the structure of actin fibers and nuclei, and the capacity of the cells to migrate, were examined. The concentrations selected for the in vitro study were 10, 25, 50, 75, and 100 μM, equivalent in milligrams to 4.4, 11.1, 22.2, 33.3, and 44.4 mg of tetracycline and 3.4, 8.7, 17.4, 26.2, and 34.9 mg of ampicillin, respectively. According to the formula described by Levy, an estimated calculation was made in order to correlate the concentrations used in vitro with those used in vivo [52]. Accordingly, after the administration of a dose of 500 mg of tetracycline, the plasma concentration is approximately 5 mg/mL [53]. Meanwhile, in the case of ampicillin, the plasma concentration is approximately 23.1 mg/mL [54]. Consequently, the concentrations selected for the in vitro study were based on the plasma concentrations achieved in vivo.

In current practice, tetracyclines family, which include tetracycline, doxycycline, and minocycline, are used primarily due to their antibacterial properties. Antibacterial activity is primarily achieved by binding to small ribosomal subunits and blocking the attachment of aminoacyl-tRNA to site A in the ribosome [55]. The first mention of the possibility of tetracycline having an antitumor effect was made in the 1980s [27]. There was a correlation between this anti-tumor effect and the inhibition of mitochondrial protein synthesis, thus resulting in a decrease in mitochondrial energy generation capability in cells with increased proliferation, thus affecting cellular proliferation [56]. Further studies have demonstrated that tetracycline induces various effects related to mitochondrial damage, including cytochrome c-mediated apoptosis of cells and diminution in respiration and membrane potential [57,58,59]. The use of tetracycline in the treatment of head and neck cancers has been investigated because it may prevent surgical site infections after surgery for oral cancer. Based on the findings of the study conducted by Funahara and colleagues, the topical administration of tetracycline 48 h following surgical intervention is effective at preventing infection [60]. Tetracycline analogues have recently been studied at the level of several types of tumor cells. As a result, doxycycline, minocycline, and chemically modified tetracycline-3 were examined in human acute myeloid leukemia cells—HL-60 for their antitumor properties. Researchers found that all three tetracyclines reduced cell viability, resulting in morphological changes characteristic of apoptosis, data that are in agreement with our results [58]. Doxycycline has also been shown to inhibit the proliferation of colon cancer cells by inhibiting matrix metalloproteinases [61]. Studies using different tumor cell lines, such as cervical cancer and breast cancer cells, have confirmed the cytotoxic and antiproliferative effects of doxycycline in vitro [62,63]. The antitumor properties of minocycline, another tetracycline representative, have been extensively studied. Consequently, it was found to be a promising antitumor agent in the case of ovarian cancer, inhibiting the proliferation of cells and preventing the formation of tumor colonies. These actions are due to the suppression of interleukin-6 (IL-6) expression and the transforming growth factor-β-activated-kinase-1 (TGF-β1-TAK1-IκB) signaling pathway [64,65]. Minocycline has also been evaluated and proven effective against other tumor cell lines, including breast cancer cells [66]. Tigercycline is another tetracycline that has been studied for its antitumor properties. There was a significant antitumor effect observed at the level of different tumor cell lines, including myeloid leukemia, non-small cell lung cancer, and negative breast cancer [67]. Accordingly, the present study was designed to evaluate the parent compound of the tetracycline class. The results indicated that by stimulating cells for 72 h with five concentrations of tetracycline (10, 25, 50, 75, and 100 μM), there was a dose-dependent reduction in cell viability. Meanwhile, morphological changes characteristic of the apoptosis process were observed. Additionally, tetracycline altered the structure of the nucleus and the organization of actin fibers, which are characteristic of apoptosis in cells. Additionally, the results showed that the compound had a strong effect on inhibiting cell migration. Based on the findings of Hirasawa’s study, doxycycline inhibits autophagy as well as cell viability in pharyngeal carcinoma cells—Detroit-562 [68]. As part of their study, Gouzos et al., investigated the impact of antibiotics from the tetracycline class on the health of healthy cells using primary human nasal epithelial cells and primary fibroblasts. According to the study, doxycycline contributes to the healing of chronic wounds by encouraging the migration of fibroblasts and epithelial cells. Tetracyclines exhibit a positive effect on wound healing due to their immunomodulatory and anti-inflammatory properties caused by their inhibition of matrix metalloproteinases (MMP) [69]. Similarly, the long-term incubation of the human retinal pigment epithelial cells—ARPE-19 with higher doses of tetracycline led to an increase in cell viability and a decrease in reactive oxygen species, emphasizing the beneficial effect of tetracycline on healthy cells due to the inhibition of matrix metalloproteinase activity [70]. Furthermore, Ishikawa and colleagues evaluated the effects of tetracycline, doxycycline, and minocycline on a human keratinocyte cell line, HNEK. Results showed that tetracycline inhibited the production of interleukin-8, which had an anti-inflammatory effect on keratinocytes [71].

**Figure 8:**
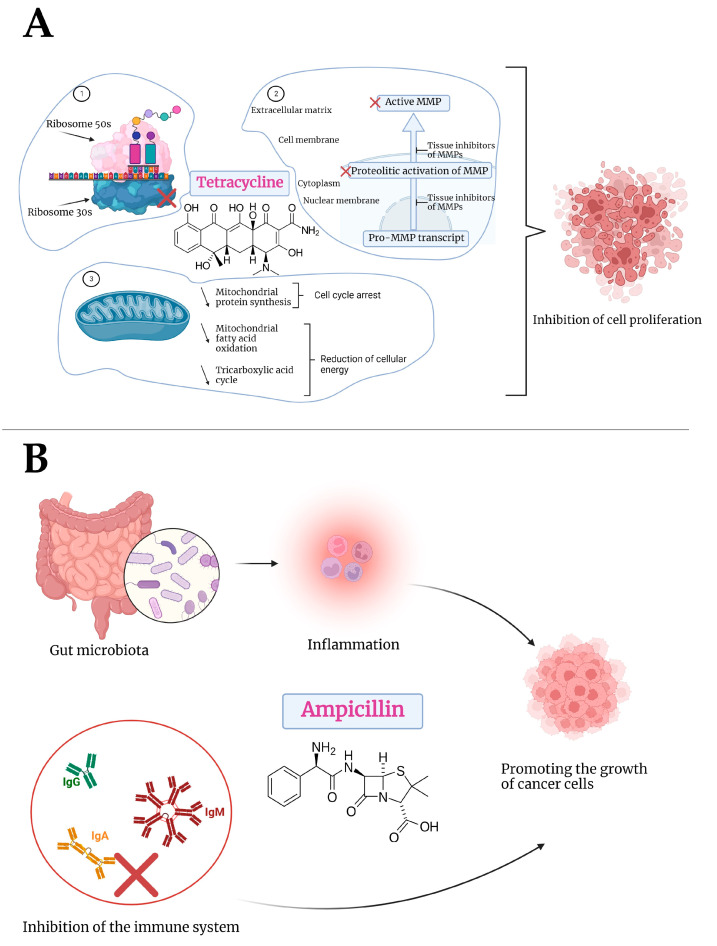
Schematic representation of the mechanism of action of tetracycline (**A**) and ampicillin (**B**). (**A**) 1. Tetracycline binds to the ribosomal site, thereby blocking the synthesis of proteins. A similar process can be observed in mitochondrial ribosomes, with tetracycline inhibiting mitochondrial protein synthesis. 2. Tetracycline inhibits MMPs by acting at the transcriptional and protein initiation levels, thereby inhibiting cell migration and proliferation as well as cell adhesion. 3. Tetracycline inhibits mitochondrial protein synthesis with energy shortage and cell cycle arrest. The mitochondrial oxidation of fatty acids decreases at the same time, resulting in the down-regulation of the tricarboxylic acid cycle and a decrease in cellular energy. (**B**) Ampicillin affects the gut microbiota, which leads to systemic inflammation and favors the growth of tumor cells. Additionally, ampicillin affects the immune system by decreasing the level of IgA, IgG, and IgM, resulting in the development and proliferation of tumor cells. Created with BioRender.com (accessed on 8 September 2022).

Additionally, in the present study, the effect of ampicillin was also examined. In this case, the results indicated a different response in Detroit-562 cells compared with tetracycline. Therefore, Ampicillin did not exhibit a significant cytotoxic effect; in fact, at concentrations of 10, 25, and 50 μM, cell viability increased compared to untreated control cells. Meanwhile, the concentrations of 75 and 100 μM resulted in a slight decrease in viability, but the decrease was not significant, with values of approximately 95% and 94%, respectively. The morphology of cells, as well as the structure of the nucleus and the organization of actin fibers, did not demonstrate any significant differences from those seen in control cells. The results of an in vivo study on a murine model indicated that the administration of ampicillin changes the microbiota, which results in an increase in the size of breast cancer tumors, causing a protumoral response [72]. According to a study conducted between 1989 and 2012, penicillins, especially ampicillin, may increase the risk of colorectal cancer [73]. The use of penicillins, including ampicillin, in the treatment of head and neck cancer, was examined by Iocca and colleagues in a systematic review, which found that ampicillin was one of the most effective antibiotics for preventing postoperative infections [74]. In a similar study, Veve et al. evaluated the effectiveness of different antibiotics in preventing surgical site infections in patients with head and neck cancer. A combination of ampicillin and sulbactam was one of the most effective antibiotics [75]. On the other hand, the use of antibiotics during the treatment of head and neck cancers was evaluated by Nenclares et al. Among the most commonly used antibiotics were penicillins and penicillin analogues, which were used primarily in the prevention of local reactions in the dentition caused by radiotherapy. Among the findings of the study, the researchers identified antibiotic treatment as a risk factor for reducing progression-free survival, overall survival, and disease-specific survival [5]. Khatoon and colleagues examined the impact of silver nanoparticles with ampicillin on healthy human keratinocytes. As a result of the study, it was determined that these formulations have no cytotoxic effects on the human keratinocyte cell line HaCaT [76]. Regarding its mechanism of action, ampicillin inhibits enzymes present in the bacterial cell walls responsible for the synthesis of peptidoglycan layer of bacterial cell walls by binding to the penicillin-binding proteins. In light of this, ampicillin does not cause unfavorable effects on human cells because peptidoglycans are absent [77]. A previous study evaluated the effects of these two antibiotics on colorectal carcinoma cells—HT-29—observing a similar effect to that reported in the present study. After 72 h of stimulation with five concentrations (10, 25, 50, 75, and 100 μM), tetracycline strongly inhibited cell viability, whereas ampicillin stimulated cell proliferation [78]. A possible explanation for the effect of ampicillin on tumor cell proliferation was provided by Boursi and colleagues, who noted that penicillin inhibited the immune system and decreased IgA, IgM, and IgG levels [79]. In addition, beta-lactam antibiotics can cause damage to the microbiota, which can lead to systemic inflammation, promoting the growth of tumor cells [80]. Additionally, tetracycline’s cytotoxic effects may result from a variety of possible biological mechanisms, including: (i) inhibition of mitochondrial protein synthesis [81]; (ii) inhibition of matrix metalloproteinases [82]; (iii) angiogenesis damage [83]; (iv) eradicating cancer stem cells [84]; and (v) increasing the sensitivity of tumor cells to radiotherapy through down-regulation of DNA-dependent protein kinase [85]. Figure 8 shows the mechanisms of action of tetracycline and ampicillin.

According to these results, the present study’s findings are consistent with those obtained in the previous study. As far as we know, no causal relationship has been established between pharyngeal cancer and ampicillin and tetracycline use. This is one of the novel aspects of this study.

## 5. Conclusions

The purpose of this study was to evaluate the effectiveness of two broad-spectrum antibiotics—tetracycline and ampicillin—at the level of pharyngeal carcinoma cells—Detroit-562. The results indicated that the two antibiotics had different effects. Accordingly, tetracycline has a concentration-dependent cytotoxic effect, characterized by a decrease in cell viability as well as morphological changes characteristic of apoptosis (condensation of the nucleus and actin fibers, appearance of apoptotic bodies). In contrast, ampicillin caused a slight decrease in cell viability at higher concentrations, but insignificant compared to control cells, whereas lower concentrations caused a stimulation of cell proliferation. In conclusion, tetracycline has been shown to be a potential antitumor agent, but further studies are necessary to clarify its biological mechanism of action and determine its safety profile. In terms of ampicillin, it appears to have the potential to stimulate the proliferation of tumor cells, but detailed studies are also necessary to accurately determine the risks associated with its use.

Among the main novelty aspects of the present study is the evaluation of two broad-spectrum antibiotics, tetracycline, and ampicillin, at the level of pharyngeal carcinoma cells—Detroit-562. To our knowledge, the two antibiotics have not yet been evaluated for their cytotoxic activity, cell migration, cell morphology, and influence on the structure of the nucleus and actin fibers of Detroit-562 cells. Considering that two broad-spectrum antibiotics, which are widely used worldwide, were investigated in the present study, the impact they have on tumor cells is of great interest. Antibiotics’ impact on the microbiota and its implications for the development and progression of cancer is a real focus of today’s research. In order to fully understand how tetracycline and ampicillin affect the microbiota in the throat and how this microbiota contributes to pharyngeal cancer, further studies are necessary.

## Data Availability

Not applicable.
